# Cannabidiol Enhances the Therapeutic Effects of TRAIL by Upregulating DR5 in Colorectal Cancer

**DOI:** 10.3390/cancers11050642

**Published:** 2019-05-09

**Authors:** Jung Lim Kim, Bo Ram Kim, Dae Yeong Kim, Yoon A. Jeong, Soyeon Jeong, Yoo Jin Na, Seong Hye Park, Hye Kyeong Yun, Min Jee Jo, Bu Gyeom Kim, Han Do Kim, Dae Hyun Kim, Sang Cheul Oh, Sun Il Lee, Dae-Hee Lee

**Affiliations:** 1 Division of Oncology, Department of Internal Medicine, Korea University College of Medicine, Korea University Guro Hospital, Seoul 08308, Korea; clickkjl@naver.com (J.L.K.); ilovewish777@naver.com (B.R.K.); limlab7@gmail.com (S.J.); sachoh@korea.ac.kr (S.C.O.); 2 Graduate School of Medicine, Korea University College of Medicine, Seoul 02841, Korea; derrickdyblue22@gmail.com (D.Y.K.); leomi2614@naver.com (Y.A.J.); wing1278@naver.com (Y.J.N.); psh3938@hanmail.net (S.H.P.); katecoco@hanmail.net (H.K.Y.); minjeeyoyo@nate.com (M.J.J.); qnrua10047@naver.com (B.G.K.); 3 Kaiyon Bio Tech Co., Ltd, 226 Gamasan-Ro, Guro-gu, Seoul 08308, Korea; howard@kaiyonbiotech.com (H.D.K.); dkim238@kaiyonbiotech.com (D.H.K.); 4 Department of Surgery, Korea University Guro Hospital, Korea University College of Medicine, Seoul 08308, Korea

**Keywords:** cannabidiol, TNF-related apoptosis-inducing ligand, death receptor 5, endoplasmic reticulum stress

## Abstract

Cannabidiol, a major non-psychotomimetic compound derived from *Cannabis sativa*, is a potential therapeutic agent for a variety of diseases such as inflammatory diseases, chronic neurodegenerative diseases, and cancers. Here, we found that the combination of cannabidiol and TNF-related apoptosis-inducing ligand (TRAIL) produces synergistic antitumor effects in vitro. However, this synergistic effect was not observed in normal colonic cells. The levels of ER stress-related proteins, including C/EBP homologous protein (CHOP) and phosphorylated protein kinase RNA-like ER kinase (PERK) were increased in treatment of cannabidiol. Cannabidiol enhanced significantly DR5 expression by ER stress. Knockdown of DR5 decreased the combined effect of cannabidiol and TRAIL. Additionally, the combination of TRAIL and cannabidiol decreased tumor growth in xenograft models. Our studies demonstrate that cannabidiol enhances TRAIL-induced apoptosis by upregulating DR5 and suggests that cannabidiol is a novel agent for increasing sensitivity to TRAIL.

## 1. Introduction

Colorectal cancer (CRC) is the third most common cancer worldwide, with nearly 1.4 million new cases diagnosed in 2017 [1]. Treatment of colorectal cancer most commonly involves a combination of three classic strategies of oncology: surgery, radiation, and chemotherapy. Many chemotherapeutic regimens have been used clinically for the treatment of colorectal cancer, but it is necessary to continue to develop new therapeutic agents for its successful treatment.

TNF-related apoptosis-inducing ligand (TRAIL) is a type 2 membrane protein belonging to the TNF superfamily. TRAIL induces apoptosis via interaction with its corresponding death receptors (TRAIL-R1/DR4 and TRAIL-R2/DR5) [2]. TRAIL is one of the few tumor-selective agents that selectively kill cancer cells by activating signaling pathways used by the innate immune system, and it is not toxic to normal cells [3]. TRAIL has therapeutic potential for the treatment of cancer and is currently undergoing clinical phase І and ІІ trials [4,5]. Nevertheless, recombinant TRAIL has limitations, such as its short plasma half-life and cellular resistance to its apoptotic effects [6]. The mechanisms of TRAIL resistance that have been reported include downregulation of death receptors, loss of apoptotic proteins, and activation of the NF-κB and PI3K/AKT pathways [7,8,9]. Therefore, it is necessary to understand the underlying mechanisms of the resistance to TRAIL and to overcome this resistance. It is possible to increase the sensitivity to TRAIL by combining it with other anticancer agents.

**Figure 1:**
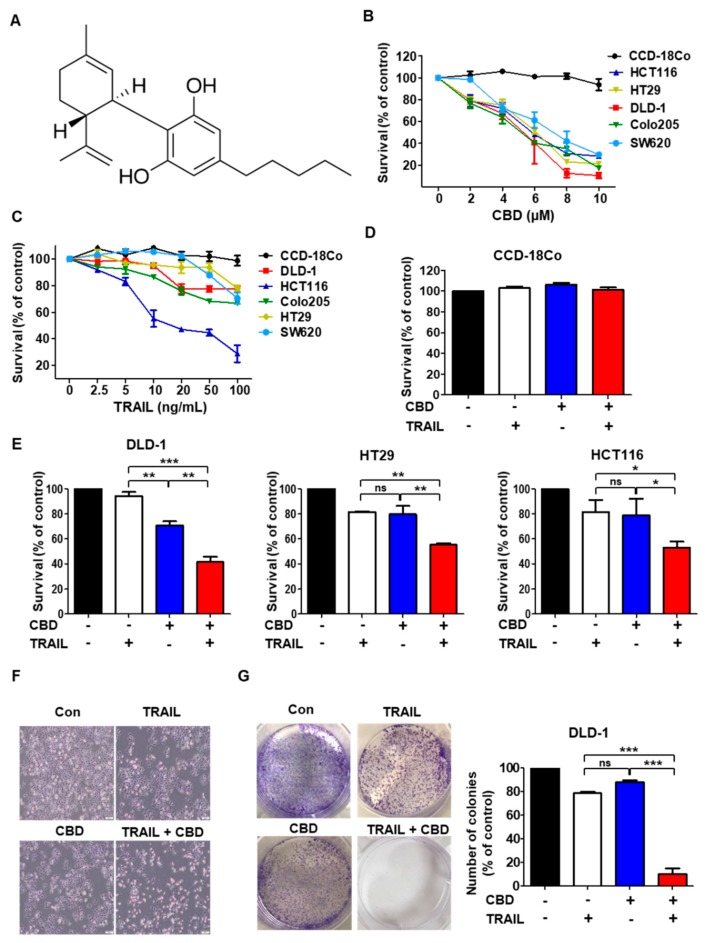
Cytotoxic assay of colorectal cancer cells after exposure to cannabidiol in combination with TNF-related apoptosis-inducing ligand (TRAIL). (**A**) Structure of cannabidiol. (**B**) Various cell lines were treated with cannabidiol for 24 h. (**C**) Cells were exposed to TRAIL for 4 h. (**D**) CCD-18Co (normal colon) cells were pretreated with or without 4 µM cannabidiol for 24 h and then with TRAIL 10 ng/mL for 4 h. Cell survival was determined (**E**) DLD-1, HT29, and HCT116 cells were treated with cannabidiol (4 μM) alone or in combination with TRAIL (DLD-1, HT29 10 ng/mL, HCT116 2.5 ng/mL). For parts B–E, cell survival was determined by the WST-1 assay. (**F**) Morphology of DLD-1 cells that were pretreated with or without 4 µM cannabidiol for 24 h and then treated with TRAIL at 10 ng/mL for 4 h. 100×. (**G**) Clonogenic assay was assessed after 7 days of cannabidiol treatment alone or combined with TRAIL and wells were stained with crystal violet at the end of the experiment. Left; Photographs of wells in a representative experiment are shown. Right; percentage inhibition of colony formation after drug treatment. Each result is presented as the mean of 3 independent experiments * *p* < 0.05; ** *p* < 0.01; *** *p* < 0.001. CBD, cannabidiol; ns, not significant.

Cannabidiol (Figure 1A), a major non-psychotomimetic compound derived from *Cannabis sativa*, is a potential therapeutic agent for a variety of diseases such as inflammatory diseases, chronic neurodegenerative diseases, and cancers. In particular, cannabidiol is being tested as a potential anticancer agent for a variety of cancers, including breast cancer, neuroblastoma, glioma, prostate cancer, and colorectal cancer [10,11,12,13]. Cannabidiol is already undergoing clinical trials for the treatment of brain tumors such as gliomas. In colorectal cancer cell lines, cannabidiol reduced cell proliferation through antagonism of cannabinoid receptor type 1 (CB1) and transient receptor potential cation channel subfamily V member 1 (TRPV1), protected DNA from oxidative damage, and increased endocannabinoid levels [14]. However, the underlying mechanism of the antitumor function of cannabidiol in solid tumor cells is not fully understood. Thus, there is interest in investigating the possible TRAIL-sensitization of cancer cells by the combined treatment of cannabidiol and TRAIL, which could enhance cell death.

The endoplasmic reticulum (ER) plays an essential role in protein synthesis, maturation, lipid synthesis, calcium homeostasis, and protein folding [10]. Intermediate ER stress acts as a defense mechanism for cell survival, but severe or prolonged ER stress may lead to the initiation of apoptosis [11,12]. Recent studies have found that anticancer agents, reactive oxygen species, hypoxia, and radiation aggravate ER-stress responses and activate ER stress-mediated apoptosis pathways in cancer [13,14]. Therefore, we hypothesized that cannabidiol activates ER stress-related apoptosis via exacerbating reactive oxygen species generation and aggravating ER-stress responses.

In the present study, we investigated whether the combination of cannabidiol and TRAIL could induce apoptosis in colorectal cancer cells. We found that cannabidiol enhanced TRAIL-induced apoptosis by increasing the expression of DR5 through ER-stress in colorectal cancer cells. Collectively, our results suggest that cannabidiol is a new agent for increasing the sensitivity to TRAIL

## 2. Materials and Methods

### 2.1. Cell Culture

The human colorectal cancer cell lines, HCT116, HT29, and DLD-1, were purchased from the American Type Culture Collection (ATCC, Manassas, VA, USA). The cells were cultured as monolayers in RPMI 1640 medium (Invitrogen, Carlsbad, CA, USA) with 10% fetal bovine serum (HyClone, Logan, UT, USA), 1 mM L-glutamine, and 26 mM sodium bicarbonate. Human normal colon (CCD18CO) and lung (BEAS-2B) cell lines were purchased from ATCC. All cell lines were grown at 37 °C in a humidified chamber with 5% CO_2_.

### 2.2. Reagents and Antibodies

Cannabidiol and VAS2870 were purchased from Sigma (St. Louis, MO, USA). TRAIL and anti-DR5 were purchased from R&D Systems (Minneapolis, MN, USA). Anti-Bak, anti-Bcl-2, anti-Mcl-1, anti-Bcl-xL, and anti-DR4 antibodies were purchased from Santa Cruz Biotechnology (Santa Cruz, CA, USA). Anti-XIAP, anti-NOXA, anti-BIM, anti-survivin, anti-Bid, anti-IRE1α, anti-phospho-IRE1α, anti-Bip, anti-GRP94, anti-ATF6, anti-eIF2α, anti-phospho-eIF2α, anti-CHOP, anti-cleaved PARP, anti-caspase-3, and anti-caspase-9 antibodies were purchased from Cell Signaling Technology (Danvers, MA, USA). The anti-actin antibody was purchased from Sigma-Aldrich (St. Louis, MO, USA). For the secondary antibodies, anti-mouse IgG horseradish peroxidase (HRP) and anti-rabbit IgG HRP were purchased from Cell Signaling Technology.

### 2.3. Western Blotting

The cells were lysed in a RIPA buffer (50 mM Tris, 150 mM NaCl, 1% Triton X-100, 0.1% SDS, and 1% sodium deoxycholate (pH 7.4)) containing a protease and phosphatase inhibitor cocktail (Sigma-Aldrich). Protein concentrations were measured using the bicinchoninic acid protein assay reagent (Thermo Fisher Scientific, Waltham, MA, USA). Equal amounts of proteins were separated by SDS-PAGE and transferred to nitrocellulose membranes (GE Healthcare Life Sciences, Little Chalfont, UK). The membranes were blocked with TBS containing 0.2% Tween 20 and 5% skim milk, incubated with primary antibodies overnight at 4 °C, and then incubated with HRP-labeled secondary antibodies. The signals were detected using X-ray film.

### 2.4. Colony Formation Assay

The cells were seeded in 6-well plates at a density of 500 cells per well and then were cultured at 37 °C. The medium was changed every three days. After one week, the cells were washed with PBS, fixed with 4% paraformaldehyde for 30 min, and then stained with crystal violet for 30 min for visualization and counting.

### 2.5. Flow Cytometry Analysis of Cell Apoptosis

The translocation of phosphatidylserine, an apoptosis marker, from the inner to the outer leaflet of the plasma membrane was detected by the binding of fluorescein isothiocyanate (FITC-conjugated annexin V. Briefly, DLD-1 cells, which had been untreated or treated with cannabidiol, TRAIL, or a combination of these two agents, were resuspended in the binding buffer provided with the Annexin V-FITC Apoptosis Detection Kit (BioBud, Seoul, Korea, Cat. No. LS-02-100). The cells were then mixed with 1.25 μL of the annexin V-7 μL fluorescein isothiocyanate (FITC) reagent and incubated for 30 min at 4 °C in the dark. The staining was then terminated and the cells were immediately analyzed by flow cytometry (Beckman Coulter, CA, USA).

### 2.6. Quantitative Reverse Transcription PCR (qRT-PCR)

Total RNA was extracted by using TRIzol reagent (Life Technologies, CA, USA). The amplification of transcripts was performed using a reverse transcriptase PCR kit (Life Technologies). qPCR was performed on an Applied Biosystems 9700 thermal cycler using gene-specific oligonucleotide primers and Taqman™ probes (Applied Biosystems, CA, USA). The primers and Taqman™ probes were as follows: GAPDH (Hs99999905_m1) and DR5 (Hs00366278_m1). The mRNA expression was normalized to that of GAPDH. The ΔΔCT method was used to assess the relative mRNA expression level.

### 2.7. Small Interfering RNA (siRNA)

DR5 siRNA, CHOP siRNA, and negative control siRNA were purchased from Santa Cruz Biotechnology. The cells were transfected with siRNA oligonucleotides using the Lipofectamine RNAi Max reagent (Invitrogen) according to the manufacturer’s instructions.

### 2.8. Immunofluorescence Staining

The cells were grown on glass coverslips and were fixed with 3.7% formaldehyde for 15 min, followed by permeabilization with 0.5% Triton X-100 for 15 min at room temperature. The cells were then blocked for 1 h with 3% bovine serum albumin and probed with primary antibodies overnight at 4 °C. The cells were washed and then incubated with Alexa Fluor^®^ 594-conjugated secondary antibody (Molecular Probes, Eugene, OR, USA) or FITC-conjugated secondary antibody (Sigma-Aldrich). The nuclei were counterstained with 4′,6-diamidino-2-phenylindole (DAPI). The cells were mounted with VECTASHIELD mounting medium (Vector Laboratories, Burlingame, CA, USA) and visualized by fluorescence microscopy.

### 2.9. ROS Measurement (DCFH-DA Assay)

ROS levels were measured using dichloro-dihydro-fluorescein diacetate (DCFH-DA). Cells were incubated for 30 min with 20 µM DCFH-DA, and then washed with PBS. Cells were fixed with 3.7% formaldehyde for 15 min at room temperature. Fluorescence intensity was measured using a flow cytometer or fluorescence microscopy.

### 2.10. Tumor Xenograft Experiment

All animal experiments were carried out in accordance with animal care guidelines approved by the Korea University Institutional Animal Care and Use Committee (IACUC, KOREA-2018-0081). Four-week-old female BALB/c nude mice were acquired from Orient Bio (Kyonggi, Korea) and housed in a specific, pathogen-free environment. The animals were acclimated for one week prior to the study and were provided unlimited access to food and water. DLD-1 cells (3 × 10^6^) in 100 µL of culture medium were mixed with 20 µL of Matrigel and implanted subcutaneously into five-week-old BALB/c nude female mice. The tumor size was measured every two days.

### 2.11. Immunohistochemistry (IHC) Staining and Scoring

Sections of formalin-fixed, paraffin-embedded tumor specimens were deparaffinized in xylene and hydrated in a graded alcohol series. Endogenous peroxidase was blocked using 3% hydrogen peroxide in distilled water for 15 min, and antigen retrieval was performed by heating at 100 °C for 20 min. The tissue slides were incubated with a universal blocking solution (BioGenex, Fremont, CA, USA) for 15 min at room temperature, and then incubated at 4 °C overnight with primary antibodies. The antibodies, catalog numbers, and dilutions used in this method are listed in Table 1. The samples were incubated with peroxidase-conjugated anti-goat IgG for 1 h at room temperature. IHC reactions were visualized by 3-3′-diaminobenzidine staining using the EnVision+ system (Dako, CA, USA).

### 2.12. Terminal Deoxyribonucleotidyl Transferase-Mediated dUTP Nick End Labeling (TUNEL) Staining

TUNEL staining was performed using the In Situ Cell Death Detection Kit, TMR red (ROCHE; Cat. No. 12156792910, Basel, Switzerland) according to the manufacturer’s instructions.

### 2.13. Combination Index and Statistical Analysis

To determine whether the cytotoxic interactions of cannabidiol and TRAIL were synergistic, additive, or antagonistic in colorectal cancer cells, drug effects were examined using the combination index (CI) method of Chou and Talalay. GraphPad InStat 6 software was used for all statistical analyses (GraphPad Software, Inc., La Jolla, CA, USA). For comparisons among groups, one-way ANOVA followed by Tukey’s post-hoc tests was used. To determine significance between two groups, an unpaired t-test was used. A *p*-value of less than 0.05 was considered significant.

## 3. Results

### 3.1. Cannabidiol Enhances TRAIL-Induced Cell Death in Human Colorectal Cancer Cells

First, to investigate the role of cannabidiol (Figure 1A) in cell death, we performed an MTT assay. Cannabidiol mediated a concentration-dependent reduction in colorectal cancer cell viability, but not in normal primary colon cells (CCD18-Co) (Figure 1B). Only a few colorectal cancer cell lines are sensitive to cell death mediated by TRAIL (Figure 1C). We performed a combination index (CI) for selecting the most effective concentration using the Compusyn software. As shown in Supplementary Figure S1, the combination of 4 μM cannabidiol and 10 ng/mL TRAIL showed the best combination effect (Supplementary Figure S1). Thus, in the first set of experiments to assess whether cannabidiol enhances cell death induced by TRAIL, we performed trypan blue staining. The combination of TRAIL and cannabidiol significantly enhanced cell death in colorectal cancer cell lines (DLD-1, HT29, and HCT116) (Figure 1E), but normal primary colon cells (CCD18-Co) were not affected (Figure 1D). Next, the effect of combined TRAIL and cannabidiol on DLD-1 cell morphology was observed under a light microscope. The combination of TRAIL and cannabidiol altered cell morphology compared with that of control cells or cells treated with either reagent individually (Figure 1F and Supplementary Figure S2A,B). The colony-forming ability of the cells was decreased with the combination of TRAIL and cannabidiol compared with that of control cells or cells treated with either reagent individually (Figure 1G). These results indicate that cannabidiol enhances TRAIL-induced cell death in colorectal cancer cells.

### 3.2. Combination of TRAIL and Cannabidiol Induces Apoptosis in Colorectal Cancer Cells

**Figure 2:**
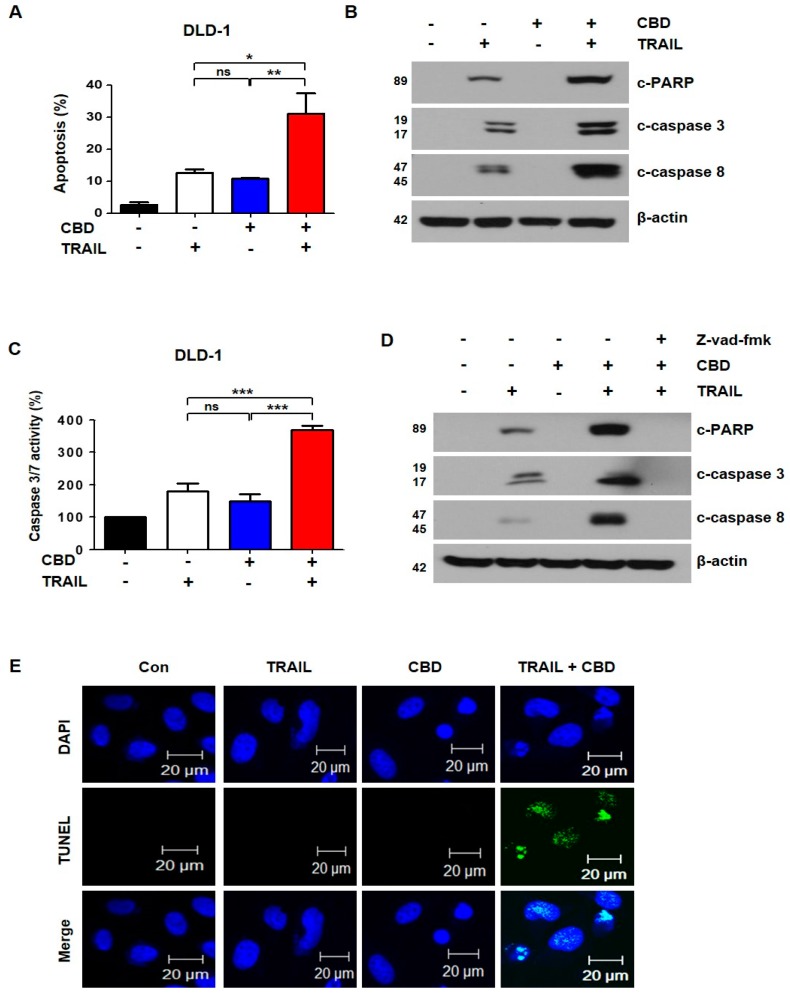
Effect of combined cannabidiol and TRAIL on TRAIL-induced apoptosis of DLD-1 cells. (**A**) DLD-1 cells were treated with 4 μM cannabidiol and/or 10 ng/mL TRAIL for 24 h. Flow cytometry using double staining with annexin V and PI was performed. Bar graphs representing the proportion of apoptotic cells are shown. Data are expressed as means ± SEM. (**B**) Effect of combined treatment with cannabidiol and TRAIL on levels of apoptosis-associated proteins. (**C**) Caspase3/7 activities were measured by ELISA-based luminescence assays following treatment with cannabidiol and/or TRAIL. (**D**) DLD-1 cells were treated with cannabidiol and/or TRAIL in the absence or presence of 25 μM z-VAD-fmk for 24 h. PARP, caspase-3, caspase-8, and β-actin protein expression levels were determined by western blot. β-actin was used as a loading control. (**E**) Terminal deoxyribonucleotidyl transferase-mediated dUTP nick end labeling (TUNEL) assay was used to evaluate apoptosis in DLD-1 cells. Cells were pretreated with or without 4 µM cannabidiol for 24 h and then exposed to TRAIL at 10 ng/mL for 4 h. * *p* < 0.05; ** *p* < 0.01; *** *p* < 0.001. CBD, cannabidiol; ns, not significant.

To investigate whether the cell death induced by the combination of TRAIL and cannabidiol was apoptosis, we performed Annexin V/propidium iodide (PI) staining with fluorescence-activated cell sorting (FACS) analysis. The combination of TRAIL and cannabidiol significantly increased apoptosis in DLD-1, HT29, and HCT116 cells (Figure 2A and Supplementary Figure S2C,D). Next, we studied the activation of PARP, caspase-3, and caspase-8. The activation of these proteins was increased with the combination of TRAIL and cannabidiol (Figure 2B and Supplementary Figure S2E,F). As shown in Figure 2C, these results were also confirmed with caspase-3 and caspase-7 activation. To determine whether apoptosis by the combination of reagents depends on caspase activation, we pretreated the cells with z-VAD-fmk, a pan-caspase inhibitor. As expected, the activation of PARP, caspase-3, and caspase-8 by the combined treatment was decreased by z-VAD-fmk (Figure 2D). We also confirmed apoptosis by the combined reagents using the TUNEL assay (Figure 2E). Therefore, our results suggest that the combination of TRAIL and cannabidiol induces caspase-dependent apoptosis.

### 3.3. Cannabidiol Increases TRAIL-Induced Apoptosis by Upregulating DR5

**Figure 3:**
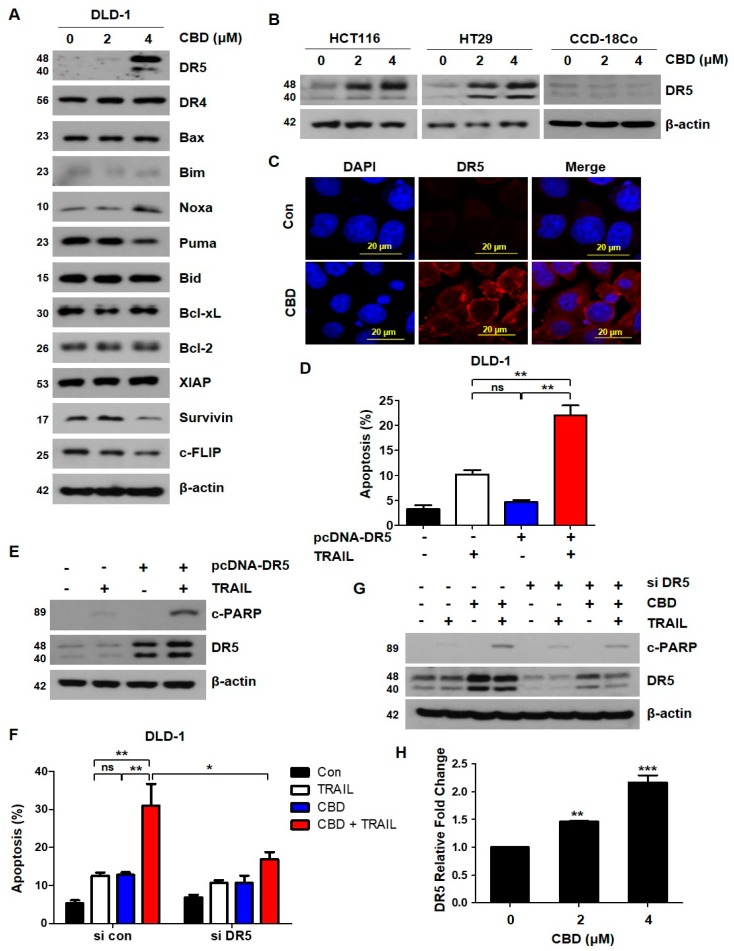
Cannabidiol induces DR5 expression in DLD-1 cells. (**A**) DLD-1 cells were treated with the indicated concentrations of cannabidiol for 24 h and the protein levels of DR4, DR5, proapoptotic, and antiapoptotic proteins were examined by western blot. β-actin was used as a loading control. (**B**) HCT116, HT29, and CCD-18Co cells were treated with the indicated cannabidiol concentrations for 24 h. Cell lysates were analyzed by western blotting using the anti-DR5 antibody. (**C**) Immunofluorescence of DR5 (red) and nucleus (blue) in DLD-1 cells exposed to cannabidiol (4 μM) for 24 h by confocal microscopy. (**D**) Plasmid-transfected, DR5-overexpressing cells were treated with 10 ng/mL TRAIL for 4 h. Bar graphs representing the proportion of apoptosis cells as assessed by flow cytometry are shown. Data are expressed as the means ± SEM. (**E**) Protein levels of PARP, DR5, and β-actin were determined by western blotting. β-actin was used as a loading control. (**F**) DLD-1 cells were transiently transfected with control siRNA (si con) or DR5 siRNA (si DR5). Twenty-four hours after transfection, cells were pretreated with or without 4 µM cannabidiol for 24 h and then treated with 10 ng/mL TRAIL for 4 h. Apoptosis was measured by flow cytometry using double staining with annexin V and PI. (**G**) Protein levels of PARP, DR5, and β-actin were determined by western blotting. (**H**) DLD-1 cells were treated with the indicated concentrations of cannabidiol for 24 h. mRNA levels of DR5 were determined by qRT-PCR. * *p* < 0.05; ** *p* < 0.01; *** *p* < 0.001. CBD, cannabidiol; ns, not significant.

To further determine the mechanisms underlying cannabidiol -mediated TRAIL effects, we examined apoptosis-related proteins, such as proapoptotic proteins, antiapoptotic proteins, and death receptors. As shown in Figure 3A, cannabidiol slightly reduced expression of survivin and c-FLIP and markedly increased DR5 expression in DLD-1 cells, but DR4 expression was unchanged (Figure 3A). These results were also observed in HCT116 and HT29 cells (Figure 3B). but DR5 expression of normal primary colon cells (CCD18-Co) was not changed (Figure 3B). The increase in DR5 was confirmed by immunofluorescence (Figure 3C). To determine whether DR5 is an important factor for TRAIL sensitivity, we transfected the plasmid, pcDNA-DR5, into DLD-1 cells. Overexpression of DR5 increased TRAIL-induced apoptosis (Figure 3D,E). In contrast, knockdown of DR5 decreased cannabidiol-induced TRAIL sensitivity (Figure 3F,G).

### 3.4. Cannabidiol Induces Activation of the ER Stress-CHOP Pathway

**Figure 4:**
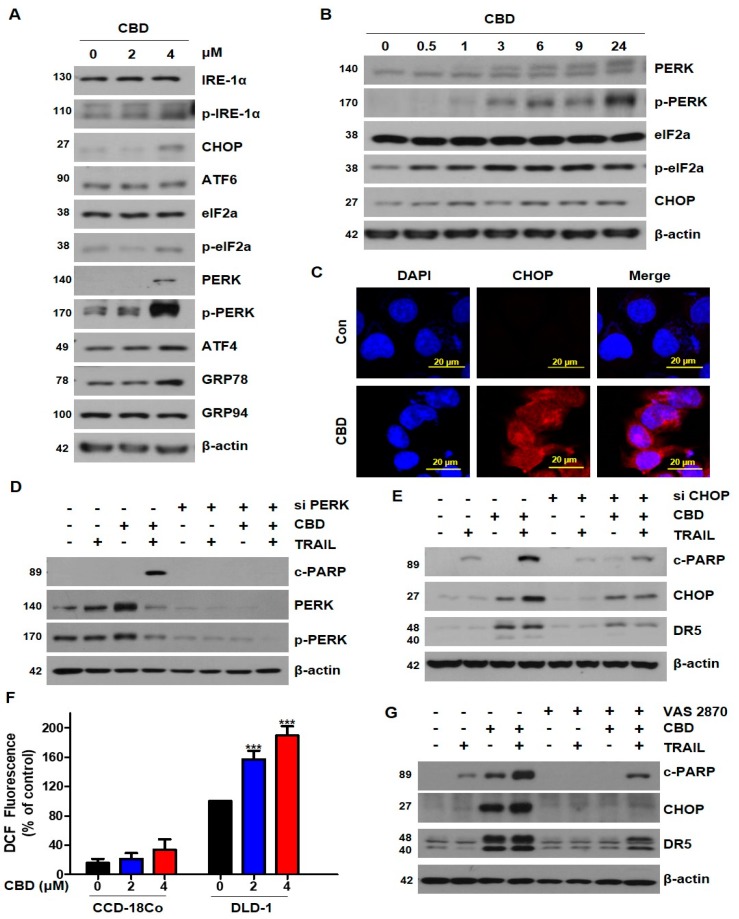
Cannabidiol induces ER stress in DLD-1 cells. (**A**) DLD-1 cells were treated with the indicated concentrations of cannabidiol for 24 h and expression levels of ER stress markers were analyzed by western blot. β-actin was used as a loading control. (**B**) Cells were treated with 4 µM cannabidiol for the indicated times. ER stress-related protein expression levels were determined by western blot. (**C**) Immunofluorescence of CHOP (**red**) and nucleus (**blue**) in DLD-1 cells exposed to cannabidiol (4 μM) for 24 h by confocal microscopy. (**D**) DLD-1 cells were transiently transfected with control siRNA and PERK siRNA. Twenty-four hours after transfection, cells were pretreated with or without 4 µM cannabidiol for 24 h and then treated with or without 10 ng/mL TRAIL for 4 h. Protein levels of PARP, PERK, p-PERK, and β-actin were determined by western blotting. (**E**) Cells were transiently transfected with control siRNA or CHOP siRNA and then treated with cannabidiol and/or TRAIL. PARP, CHOP, DR5, and β-actin protein expression levels were determined using western blot. (**F**) ROS production was assessed by DCFH-DA, following 0.5 h pre-treatment with 2, 4 μM of cannabidiol in CCD18-Co and DLD-1 cells. (**G**) Cells were pre-treated with 30 µM VAS 2870 for 30min and then treated with cannabidiol and/or TRAIL. *** *p* < 0.001. CBD, cannabidiol.

To examine whether induction of DR5 by CBD was regulated at the level of transcription, we performed qRT-PCR. As shown in Figure 3H, treatment of DLD-1 cells with cannabidiol concentration-dependently increased DR5 mRNA. Because CHOP is known as an upstream factor of DR5 [15], we tested whether cannabidiol affected ER stress. We found that cannabidiol increased DR5 via phosphorylation of protein kinase RNA-like ER kinase (PERK)-CHOP (Figure 4A). As shown in Figure 4B, cannabidiol substantially increased the PERK, eIF2α, and CHOP from 0.5 h to 24 h. The increase in CHOP induced by cannabidiol was also observed by immunofluorescence (Figure 4C). To verify whether the induction of ER stress by cannabidiol occurred via PERK, we inhibited PERK using PERK siRNA. We found that cannabidiol-induced TRAIL apoptosis was suppressed by PERK siRNA (Figure 4D). Additionally, knockdown of CHOP decreased the combined effect of TRAIL and cannabidiol (Figure 4E). Previous studies reported that cannabidiol induced ROS generation [15]. ROS is known to associate with ER stress. To investigate ROS generation induced by cannabidiol, we measured the ROS by DCFH-DA. As shown in Figure 4F, cannabidiol enhanced ROS induction. However, there is no effect on the production of ROS in normal colon cells (CCD-18Co) (Figure 4F). To demonstrate that ROS generation is responsible for the activation of ER stress induced by CBD, we treated VAS 2870, a NOX inhibitor. As shown in Figure 4G, CBD-induced DR5 and CHOP was effectively blocked on co-treated with VAS 2870. Our results indicate that CBD increases DR5 by activating ROS-PERK-CHOP.

### 3.5. Cannabidiol Enhances TRAIL-Induced Apoptosis In Vivo

**Figure 5:**
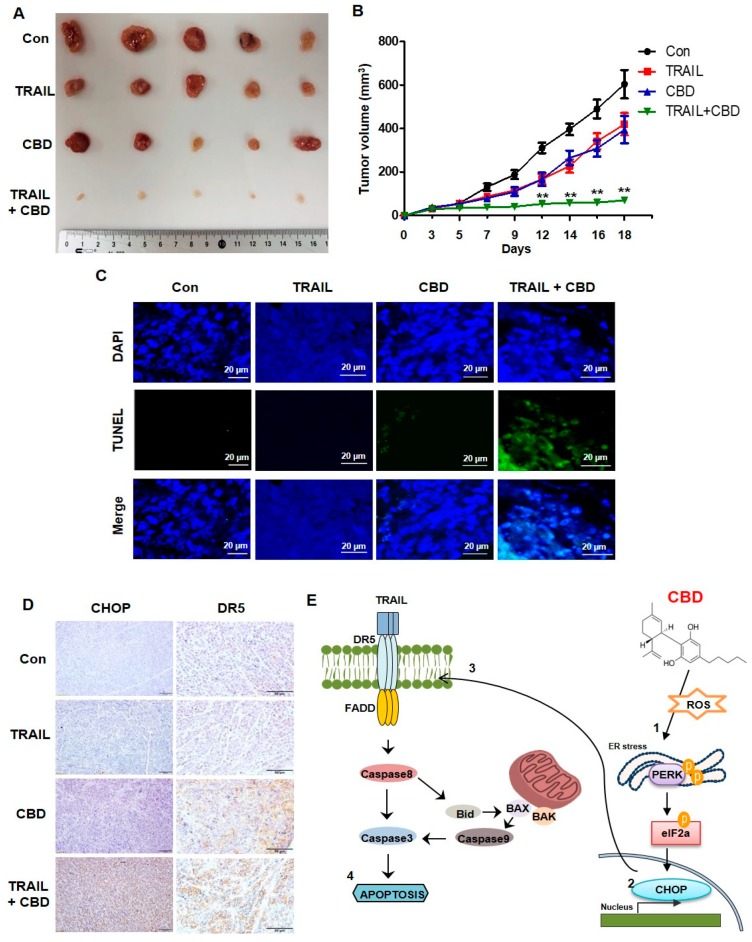
Combined antitumor effects of cannabidiol and TRAIL on colorectal cancer in vivo (**A**,**B**) DLD-1 cells were inoculated into nude mice (*n* = 5 per group) at 3 × 10^6^ per mouse subcutaneously. Mice (*n* = 5) received 4 ng/kg TRAIL and 10 mg/kg cannabidiol either alone or in combination at day 30 after tumor implantation. Representative tumors of each group are shown. (**C**) TUNEL staining of paraffin-embedded tumors. (**D**) IHC staining for CHOP and DR5 in paraffin-embedded tumors. Scale bar 50 µm. (**E**) Proposed mechanism of cannabidiol activity. CBD, cannabidiol. ** *p* < 0.01.

DLD-1 cells (3 × 10^6^) were subcutaneously injected into BALB/c nude mice. We randomly divided the mice into four groups and treated with TRAIL (4 ng/kg) and/or cannabidiol (10 mg/kg) three times per week. The combination of TRAIL and cannabidiol significantly decreased tumor growth compared to that of the control or the single-treatment groups (Figure 5A,B). Next, we performed a TUNEL assay to detect apoptosis in the tumors. As shown in Figure 5C, the number of apoptotic cells was greater in the combination group than in the other groups. We then measured DR5 and CHOP expression in the tumors using IHC. Consistent with the in vitro results, DR5 and CHOP were increased in the cannabidiol and TRAIL-cannabidiol combination group (Figure 5D). These results suggest that cannabidiol synergistically enhances TRAIL-induced apoptosis in vivo.

## 4. Discussion

TRAIL has great potential as an anticancer agent for a variety of cancers. However, some carcinomas remain resistant to TRAIL-mediated cell death by regulating apoptosis-related proteins, such as death receptors (DR4 and DR5) and anti-apoptotic proteins (IAPs, FLIP, and Mcl-1) [16]. Therefore, a new strategy for enhancing TRAIL sensitivity is required.

In the present study, cannabidiol, a new compound of natural origin, activated ER stress through upregulation of DR5 and enhanced sensitivity to TRAIL-induced apoptosis in colorectal cancer cells. Normal colonic cell (CCD-18Co) were not affected by the combined treatment of cannabidiol and TRAIL. Because the level of ROS is very low in normal cells, normal colon cell (CCD-18Co) has no response by cannabidiol (Figure 3B). Whereas, apoptotic cell death was induced in the colorectal cancer cell lines (DLD-1, HT29, and HCT116). These data indicate that the natural compound cannabidiol could be an effective TRAIL sensitizer, and the combination therapy of cannabidiol with TRAIL may be an effective treatment strategy against colorectal cancer.

Cannabinoids are lipophilic ligands for specific cell-membrane cannabinoid receptors, such as CB1 and CB2, in the G protein-coupled receptor superfamily. Cannabidiol is one of at least 113 cannabinoids identified in the cannabis plant and is known to be a major component with THC (Δ^9^-tetrahydrocannabinol). Cannabidiol has powerful anti-anxiety and antipsychotic effects. Unlike other cannabinoids, the binding of cannabidiol to the cannabinoid receptors is low affinity and it acts independently of them. Cannabidiol interacts with other receptors such as orphan G-protein coupled receptor (GPR55), peroxisome proliferator-activated receptors (PPARs), or TRPV1 [17]. Cannabidiol has shown the ability to inhibit proliferation, angiogenesis, and metastasis in various cancers, including colorectal, breast, brain, prostate, and lung cancer [12,18,19]. A recent study reported that cannabidiol-induced apoptosis of breast cancer cells by downregulation of mTOR and cyclin D1, and upregulation PPARγ [12]. Another cell-death mechanism induced by cannabidiol treatment has been reported, specifically that TNF/TNFR1 and TRAIL/TRAIL-R2 signaling are upregulated and PI3K-AKT/IKK-NF-κB signaling is suppressed in glioblastoma [20].

Recently, DR5/TRAIL-R2 and caspase-8 were shown to be universally dispensable in ER-stress-mediated apoptosis and unfolded protein response (UPR)-mediated death [21]. Moreover, the transcription factor CHOP, a downstream signaling factor of ER-stress, directly binds to the DR5 promoter and regulates DR5 expression [22]. ER stress is known to be a significant contributor to cell survival and cell death. ER stress is known to be a significant contributor to cell survival and cell death. Induction of the UPR causes ER stress and results in several pathological and physiological alterations, such as glucose depletion, hypoxia, and oxidative stress A recent study reported that cannabidiol causes ER stress-induced apoptosis in hepatic stellate cells [23]. Additionally, we found that cannabidiol induced the apoptosis of colorectal cancer cells by modulating ER stress. To the best of our knowledge, this is the first report that cannabidiol induces significant TRAIL-induced apoptosis of colorectal cancer cells, which is mediated by ER stress. In contrast, previous reports suggested that cannabidiol weakened the induction of ER stress under inflammatory conditions [24].

In summary, we conclude that the combination of cannabidiol and TRAIL is a significant potential therapy via induction of the DR5/ER stress pathway.

## 5. Conclusions

We showed that the treatment of CBD enhances TRAIL-induced apoptosis. In summary, we conclude that the combination of cannabidiol and TRAIL is a significant potential therapy via induction of the ROS/ER stress/DR5 for CRC patients.

## Figures and Tables

**Table 1:** Antibodies used for immunohistochemical staining.

Antibody	Source	Catalog Number	Dilution
DR5	Abcam	Ab8416	1:80
CHOP	novus	NB600	1:100

## Supplementary Materials

The following are available online at https://www.mdpi.com/2072-6694/11/5/642/s1, Figure S1: Combination index to cannabidiol and TRAIL CI according to cannabidiol and TRAIL in DLD-1 CI = 0.3–0.7, Synergism; CI = 0.7–0.85, Moderate Synergism; CI = 0.85–0.9, Slight Synergism, Figure S2: Effect of combined cannabidiol and TRAIL on TRAIL-induced apoptosis of HT29 or HCT116 cells.
