# Supplementary material for: Cannabidiol Enhances the Therapeutic Effects of TRAIL by Upregulating DR5 in Colorectal Cancer
Source: Cancers (Basel). 2019 May 9;11(5):642. doi: 10.3390/cancers11050642 (PMC6562873; doi:10.3390/cancers11050642)
Supplement: Supplementary file 1 [file cancers-11-00642-s001.pdf]

Supplementary Materials

Cannabidiol Enhances the Therapeutic Effects of TRAIL by Upregulating DR5 in Colorectal Cancer

Jung Lim Kim, Bo Ram Kim, Dae Yeong Kim, Yoon A. Jeong, Soyeon Jeong, Yoo Jin Na, Seong Hye Park, Hye Kyeong Yun, Min Jee Jo, Bu Gyeom Kim, Han Do Kim, Dae Hyun Kim, Sang Cheul Oh, Sun Il Lee and Dae-Hee Lee

Combination index	0.77862	0.87149	0.68285	0.67750
Description	Moderate Synergism	Slight Synergism	Synergism	Synergism
CBD (μM)	2	2	4	4
TRAIL (ng/mL)	10	20	10	20

Figure S1. Combination index to cannabidiol and TRAIL CI according to cannabidiol and TRAIL in DLD-1 CI = 0.3–0.7, Synergism; CI = 0.7–0.85, Moderate Synergism; CI = 0.85–0.9, Slight Synergism.

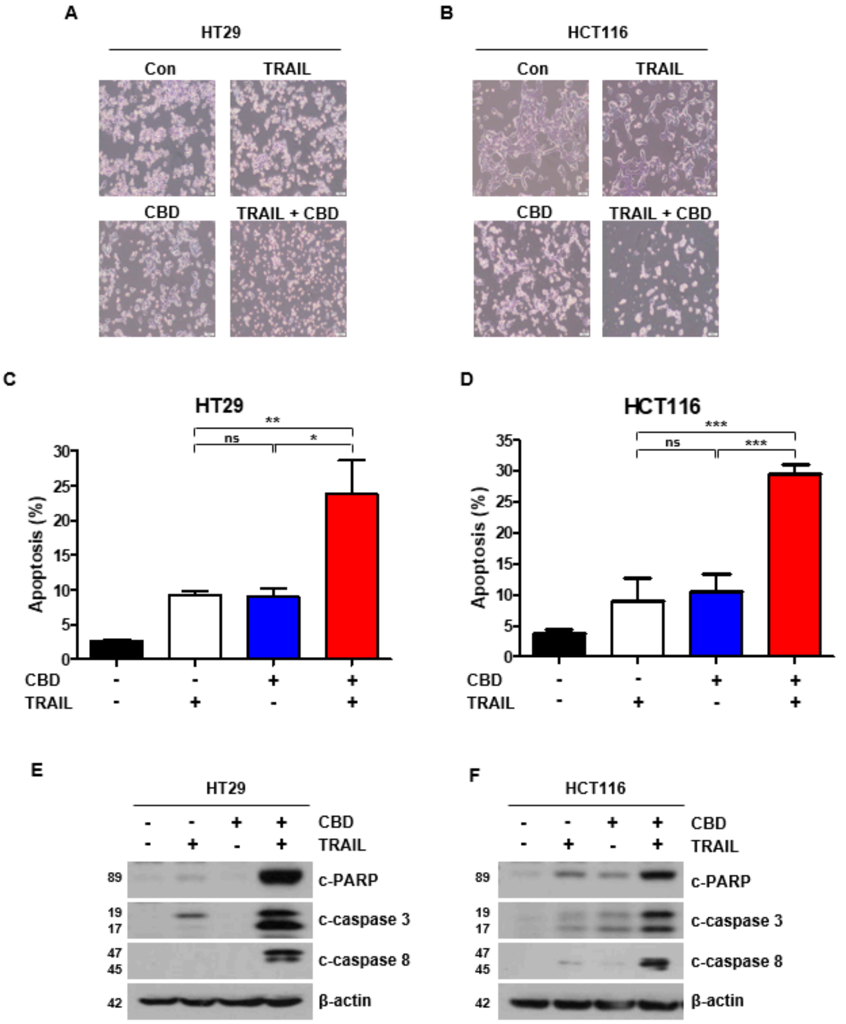

Figure S2. Effect of combined cannabidiol and TRAIL on TRAIL-induced apoptosis of HT29 or HCT116 cells. (**A**,**B**) Morphology of DLD-1 cells that were pretreated with or without 4 μM cannabidiol for 24 h and then treated with TRAIL at 10 ng/mL for 4 h. 100×. (**C**,**D**) HT29 of HCT116

cells were treated with 4 μM cannabidiol and/or 10 ng/mL TRAIL for 24 h. Flow cytometry using double staining with annexin V and PI was performed. (**E**,**F**) Effect of combined treatment with cannabidiol and TRAIL on levels of apoptosis-associated proteins. * $p < 0.05$; ** $p < 0.01$; *** $p < 0.001$. CBD, cannabidiol; ns, not significant.

© 2019 by the authors. Licensee MDPI, Basel, Switzerland. This article is an open access article distributed under the terms and conditions of the Creative Commons Attribution (CC BY) license (http://creativecommons.org/licenses/by/4.0/).
